# Testosterone-induced erythrocytosis: addressing the challenge of metabolic syndrome and widely prescribed SGLT2-inhibitor drugs

**DOI:** 10.1530/EC-24-0695

**Published:** 2025-06-21

**Authors:** Federica Tramontana, Azmi Mohammed, Yaasir H Mamoojee, Richard Quinton

**Affiliations:** ^1^ Department of Clinical & Experimental Medicine, Section of Pediatrics, University of Pisa, Pisa, Italy; ^2^ Department of Endocrinology & Metabolism, John Hopkins Aramco Healthcare, KSA, Dhahran, Saudi Arabia; ^3^ Department of Endocrinology, Newcastle Hospitals NHS Foundation Trust, The Royal Victoria Infirmary, Newcastle-upon-Tyne, UK; ^4^ Northern Region Gender Dysphoria Service, Cumbria Northumberland Tyne & Wear NHS Foundation Trust, Newcastle-upon-Tyne, UK; ^5^ Department of Metabolism Digestion & Reproduction, Imperial College London, London, UK

**Keywords:** androgens, erythrocytosis, erythropoiesis, haematocrit, haemoglobin, hypertension, metabolic syndrome, obesity, plasma volume, SGLT2-inhibitors, testosterone, thrombosis, vascular tone

## Abstract

Testosterone is the cornerstone therapy for men with hypogonadism, and also treats any associated anaemia by promoting erythropoiesis. However, excessive doses cause erythrocytosis (raised red cell mass), especially if other risk factors are present. Erythrocytosis is associated with arterial and venous thrombosis in population studies. Testosterone is now increasingly prescribed to older men with functional hypogonadism and obesity, hypertension or type 2 diabetes, who are anyway at higher risk of both erythrocytosis and thrombosis. Although short–medium term testosterone treatment in these men was not associated with adverse cardiovascular outcomes, there were more cases of pulmonary embolism. Originally envisaged as purely oral hypoglycaemic drugs, sodium-glucose cotransporter 2 inhibitors (SGLT2i) are now increasingly prescribed in chronic kidney disease (CKD), ischaemic heart disease and left ventricular impairment, irrespective of glycaemia, and the likelihood of co-prescription with testosterone is thus increased considerably. Crucially, they also increase haematocrit by promoting haematopoiesis. This review focuses on the current best evidence for managing erythrocytosis, in the context of more prevalent obesity and prescriptions of testosterone and SGLT2i in this population. It highlights the need to balance the metabolic and therapeutic benefits against the potential risks. Management strategies include re-evaluating the original treatment indication, addressing modifiable risk factors, switching to transdermal testosterone and/or reducing the testosterone dose. Venesection is not recommended, except for clonal erythrocytosis, due to its potential pro-thrombotic effects. However, combination therapy with testosterone and SGLT2s in men with anaemia of advanced CKD could augment, or even partly supersede, expensive treatment with conventional erythrocytosis-stimulating agents.

## Introduction

This review aims to inform endocrinologists about the physiology of erythropoiesis and how many of the various regulatory pathways are modulated by testosterone, which can lead to challenges managing patients who develop erythrocytosis on testosterone treatment. Although there are few data to directly link testosterone treatment with thrombosis, it is clear from general population studies that a higher haematocrit is associated with a greater rate of thrombosis, even after adjusting for other risk factors. We particularly focus on older men with metabolic syndrome and related secondary hypogonadism, who are anyway at higher risk of both thrombosis and testosterone-induced erythrocytosis. We discuss the expanding role for sodium-glucose co-transporter 2 inhibitor (SGLT2i) drugs in this population; highlighting the increasing likelihood of overlapping treatment with both testosterone and SGLT2i in these patients. We also describe the mechanisms that underpin the actions of testosterone and SGLT2is in promoting erythropoiesis, and given the increasing likelihood of patients receiving both drugs at the same time, we signpost clinicians to effective strategies for managing medication-induced erythrocytosis based on best evidence.

### Male hypogonadism and its relationship to obesity and metabolic syndrome

Male hypogonadism is characterized by low testosterone levels, subfertility and relevant clinical features (1). Although estimates of its prevalence have been strongly influenced by the diagnostic clinical criteria and biochemical cut-offs used in different studies, the European Male Ageing Study (EMAS) found that just under 4% of men aged ≥40 years fulfilled robust diagnostic criteria for hypogonadism. Of these, around 40% had pathological primary hypogonadism (PH), with raised concentrations of luteinizing hormone (LH), which was equally associated with chronological age and the burden of comorbidities (particularly obesity). The remaining 60% (defined as having late onset disease) had central hypogonadism (CH), which was overwhelmingly functional and not related to chronological ageing *per se*, but rather to the burden of comorbidities (principally obesity). In addition, nearly 10% of the overall EMAS cohort had ‘compensated’ PH, with normal testosterone but raised LH (2), a patient group that has not yet been studied in clinical trials of testosterone treatment. Both PH and compensated PH are associated with a threefold relative risk of type 2 diabetes mellitus (T2DM), irrespective of whether this is age-related or syndromic (e.g. Klinefelter syndrome or dystrophia myotonica) (1).

Many obese men present with low-normal levels of LH and testosterone, which can improve with lifestyle changes and in proportion to the weight loss achieved, highlighting the bidirectional relationship between androgens and metabolic health (3). The prevalence of metabolic syndrome is undoubtedly rising in the context of the worldwide obesity epidemic, combined with a trend to increasing longevity through better prevention and treatment of infectious diseases. Associated conditions, such as type 2 diabetes mellitus (T2DM), cardiovascular disease (CVD), cerebrovascular disease (CeVD), chronic kidney disease (CKD) and obstructive sleep apnoea (OSA), are also becoming increasingly common, and for reasons that we will outline, this has the potential to present significant challenges in managing these men as they are also being diagnosed with hypogonadism at an increasing rate.

### Testosterone treatment of obesity-associated functional hypogonadism

Testosterone is prescribed in hypogonadal males to normalise testosterone levels within the male reference range, achieve or maintain male secondary sexual characteristics, and maintain or restore sexual function, body composition, muscle strength, bone mineral density and haematopoiesis. It is also increasingly prescribed to induce and maintain virilisation in transgender males, and has additionally been advocated by some investigators as a treatment for obese older men with functional CH (or non-gonadal illness, depending on perspective), with a view to promoting metabolic health by mitigating the pro-inflammatory adipokine milieu and causing favourable changes in body composition, and improving sexual function (4, 5).

Multiple studies of testosterone treatment in men with functional CH have found improvements in metabolic parameters, including reduction in fat mass, body weight, waist–hip ratio and fasting insulin in addition to increased muscle mass. In parallel, studies of androgen deprivation therapy have shown this to induce metabolic syndrome, albeit that the gain in fat mass is predominantly subcutaneous rather than visceral. Androgens inhibit adipogenesis by blocking precursor cells from differentiating into adipocytes, limiting fat cell expansion and reducing the secretion of pro-inflammatory cytokines (e.g. leptin, IL-6, TNF-α, MCP-1, and resistin), whilst stimulating anti-inflammatory adiponectin secretion. These actions create an anti-inflammatory milieu that improves insulin sensitivity (5, 6).

Accordingly, several pilot studies of men with obesity or metabolic syndrome found a favourable impact of testosterone treatment on serum fasting glucose levels, although not HbA1c, possibly due to the effect of testosterone in increasing erythrocyte lifespan (7). Indeed, the T4DM study demonstrated the prevention or even remission of T2DM in older men at high risk, although it was not powered to detect thrombotic or cardiovascular (CV) events (8, 9). This was a randomised-controlled trial (RCT) of 12-weeks intramuscular injections of testosterone undecanoate 1 g and active lifestyle intervention versus lifestyle intervention-only in men with low or low-normal (up to 14 nmol/L) serum testosterone concentrations and Hct ≤0.50. However, T4DM followed a rigidly pharmacological paradigm that diverged from the real world prescribing of testosterone undecanoate. First, many subjects were not actually hypogonadal, and second, there was no flexibility to adjust the injection interval to avert erythrocytosis or target serum testosterone levels more precisely than just being within the reference range (low-normal at trough as per UK guidance or mid-range at the midpoint between injections as per USA guidance). Instead, the protocol envisaged the binary outcomes of either continuing 12-week injections, or if persisting erythrocytosis (or other serious adverse event) developed, triggering withdrawal from the study.

These favourable observations on T2DM risk identified in T4DM have not been substantiated by subsequent investigators. Neither TestES, a major individual patient data meta-analysis (10), nor TRAVERSE, a large prospective RCT (11, 12), found any effect of short-to-medium term testosterone treatment in older men with functional CH on either CVD or T2DM. One can only speculate as to why the much smaller T4DM study found such a marked impact of testosterone treatment on T2DM, but there are several possible explanations, including i) the unique combination with lifestyle intervention in both arms, ii) a more rigorous evaluation of glucose levels and concomitant medication, iii) the inclusion of many men who did not fulfil diagnostic criteria for hypogonadism and iv) the fixed 12-week injection-interval likely resulting in a higher overall testosterone dose. In clinical practice, many men require a longer injection interval than 12 weeks (and thus a lower dose overall) in order to achieve haemoglobin (Hb) and haematocrit (Hct) within the reference range, and serum testosterone either mid-range at the injection midpoint or low-normal at the pre-injection ‘trough’. Supporting the final hypothesis (iv) was the observation that 22% of subjects in the T4DM treatment arm compared with only 1% in placebo breached the safety trigger of Hct >054 (it is unclear whether this corresponded to the upper limit of normal for male Hct in the reference laboratory). Nevertheless, only 5% of participants discontinued testosterone therapy due to this, partly because the repeat sample (non-fasted) gave Hct ≤0.54, but also because many subjects had already received their final study treatment injection by the time of the safety trigger. Moreover, the authors emphasised the high frequency of other contributing factors and relevant comorbidities, such as OSA, in the study patients (8).

### Sodium-glucose co-transporter 2 inhibitors (SGLT2i)

The sodium-glucose co-transporter 2 (SGLT2) is a low-affinity, high-capacity transporter that is almost exclusively expressed in the brush border membrane of the S1 segment of the renal tubules and accounts for approximately 90% of glucose reabsorption. SGLT2 reabsorbs glucose in the proximal segment of the nephron via an active sodium-dependent transport process mediated by sodium-glucose co-transporters. Glucose is transported into the cell alongside Na^+^ in a 1:1 ratio via SGLT2, driven by the Na^+^ concentration gradient (13, 14).

SGLT2 inhibitors (SGLT2i) are a newer class of hypoglycaemic drugs that significantly improve CV outcomes in T2DM, CKD and heart failure. They have gained recognition for improving glycaemic control in T2DM and providing CV and renal protection in both CKD (eGFR <60 mL/min/1.73 m^2^) and left ventricular failure (ejection fraction <25% to <65%), irrespective of T2DM, supporting their routine adjunctive use in these patient groups. Moreover, their subsidiary action to promote erythropoiesis may be particularly beneficial in patients with more severe CKD and associated erythropoietin deficiency anaemia (15). However, in patients without anaemia at baseline, the combined use of testosterone and SGLT2i requires careful consideration due to the compounded risk of developing erythrocytosis and the clinical implications thereof (16).

SGLT2i improve glycaemic control by decreasing glucose reabsorption in the proximal tube and increasing glycosuria. Besides lowering blood glucose levels, SGLT2i also reduce other CV risk factors, such as blood pressure (BP), body weight, and albuminuria, providing both CV and renal protection. Several theories have been proposed to explain the molecular mechanisms underpinning these protective effects. SGLT2i reduce hyperfiltration and intraglomerular pressure by inhibiting sodium reabsorption in the proximal tubule and restoring tubuloglomerular feedback. They also lower systolic and diastolic BP by approximately 4 and 2 mmHg, respectively, by enhancing natriuresis and osmotic diuresis, resulting in reduced extracellular fluid and plasma volumes. Other beneficial mechanisms include reductions in arterial stiffness, vascular resistance, endothelial dysfunction and BP variability. In addition, SGLT2i decrease renal tubular workload and hypoxia, alongside other favourable metabolic effects (13). Natriuresis appears to be transient, with sodium reabsorption eventually returning to normal, whereas glycosuria persists long-term (14). Nevertheless, empagliflozin has been shown to be safe and effective for treating hyponatremia induced by the syndrome of inappropriate antidiuretic hormone secretion (SIADH), achieving significant increases in serum sodium levels at a safe rate of change (17).

Empagliflozin, dapagliflozin and canagliflozin are the most used agents in clinical practice, with empagliflozin having higher selectivity for SGLT2. By lowering BP, they help to slowly progress CKD and reduce CV risk in those with moderate-to-severe CKD, especially in those with albuminuria with or without T2DM (18). Other benefits in CKD include correcting renal anaemia by stimulating erythropoiesis (19) and reducing the risk of severe hyperkalaemia (K^+^ >6.0 mmol/L) without increasing the likelihood of hypokalaemia (20). In contrast, other drugs used to treat renal anaemia, such as erythropoiesis-stimulating agents (ESAs) and hypoxia-inducible factor prolyl hydroxylase domain (HIF-PHD) inhibitors, may actually increase CV risk (19). SGLT2i reduced all-cause mortality by 25% in patients with heart failure and reduced ejection fraction (21), and also reduced cardiovascular deaths and hospitalisation of patients with heart failure and preserved (or only mildly reduced) ejection fraction (22, 23).

## Haematopoiesis: physiology and pathology

### Regulatory mechanisms underpinning erythropoiesis

**Figure 1:**
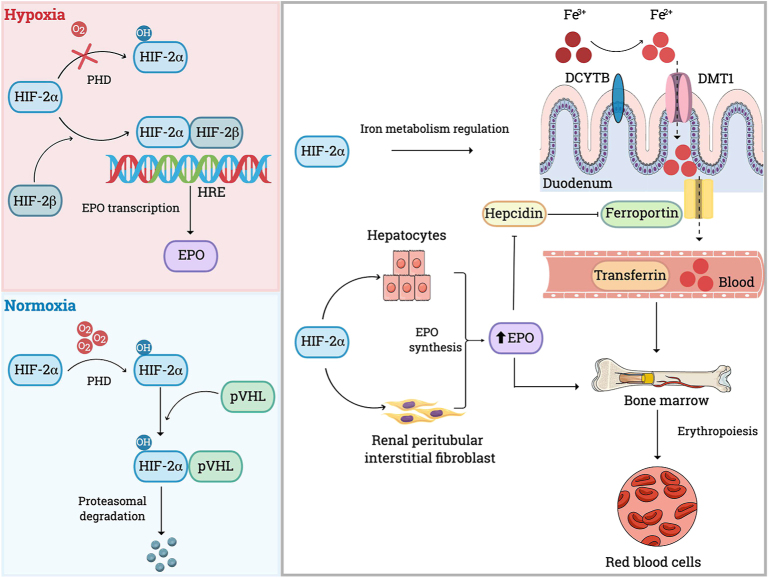
Regulation of erythropoiesis. EPO is the main regulator of erythropoiesis, and its transcription depends on tissue O_2_ levels. In normoxia, HIF2α is hydroxylated, binds to pVHL and is subsequently targeted for proteasomal degradation. In hypoxia, this degradation is decreased, allowing HIF2α to dimerize with HIF2b, and promotes EPO gene transcription. EPO is primarily produced by renal peritubular interstitial fibroblast and stimulates red blood cells production in the bone marrow. In addition to regulation EPO, HIF2α also enhances iron absorption by upregulating DCYTB (enzyme that convert Fe^3+^ in Fe^2+^) and DMT1 (facilitates Fe^2+^ uptake into cytoplasm). Iron is then exported into the bloodstream through ferroportin. EPO indirectly promotes ferroportin activity by suppressing hepcidin, a key negative regulator of iron efflux. DCYTB: duodenal cytochrome b; DMT1: divalent metal transporter 1. EPO: erythropoietin; Fe^3+^: ferric iron Fe^2+^: ferrous iron; HIF2α: hypoxia-inducible factor alpha; HIF2β: hypoxia-inducible factor beta; HRE: hypoxia response element; O_2_: oxygen molecule; OH: hydroxyl group; PHD: prolyl hydroxylase domain pVHL: von Hippel–Lindau tumour suppressor protein.

Erythropoiesis is the process of red blood cell production, primarily occurring in the bone marrow, which is regulated by erythropoietin (EPO), a glycoprotein hormone synthetized mainly in the kidney by peritubular interstitial fibroblasts, and to a lesser extent, by hepatocytes. EPO secretion is upregulated in response to systemic hypoxia, ensuring adequate oxygen delivery by increasing red cell mass (RCM). Hypoxia-inducible factors (HIFs), especially HIF2, play a central role in the regulation of EPO production and are also crucial for intestinal iron absorption and the maturation and proliferation of erythroid progenitor cells (Fig. 1).

Under normoxic conditions, HIF’s action is inhibited by von Hippel–Lindau tumour suppressor protein (pVHL), which targets HIFα subunits for polyubiquitination and proteasomal degradation. A necessary condition for the binding between HIF and pVHL is the hydroxylation of a specific proline residue of HIF, an oxygen-dependent process. Under hypoxic conditions, the activities of hydrolysation and degradation of HIF2α are reduced, enabling the HIF complex to upregulate transcription of EPO, and thus increase RBC production.

The stimulation of erythropoiesis increases iron demand in the bone marrow, and HIF2 plays a central role in iron uptake and its utilization acting directly on divalent metal transporter 1 (DMT1) and duodenal cytochrome b (DCYTB). DMT1 normally transports iron into the cell’s cytoplasm, while DCYTB reduces ferric iron (Fe^3+^) into ferrous form (Fe^2+^), facilitating iron uptake from the gut lumen into intestinal cells via DMT1. In addition, EPO inhibits the synthesis of hepcidin, a hepatocyte-derived negative regulator of iron absorption, allowing increased iron transport through ferroportin, the main extracellular iron transporter (24, 25).

Hct is a better indicator of RCM than the haemoglobin (Hb) level, which is slightly more influenced by the state of hydration (26). Hct was traditionally determined through centrifugation, but is presently estimated by automated analysers through bioimpedance analysis or laser flow cytometry. Different analysers and reagents produce different values for Hct subject to calibration by reference samples from a healthy local normal population. Accordingly, the upper limit of normality for male Hct from an accredited laboratory may range from 0.48 to 0.54, depending on how these factors combine, but will most commonly lie between 0.50 and 0.52.

### Physiological roles of testosterone in promoting erythropoiesis

**Figure 2:**
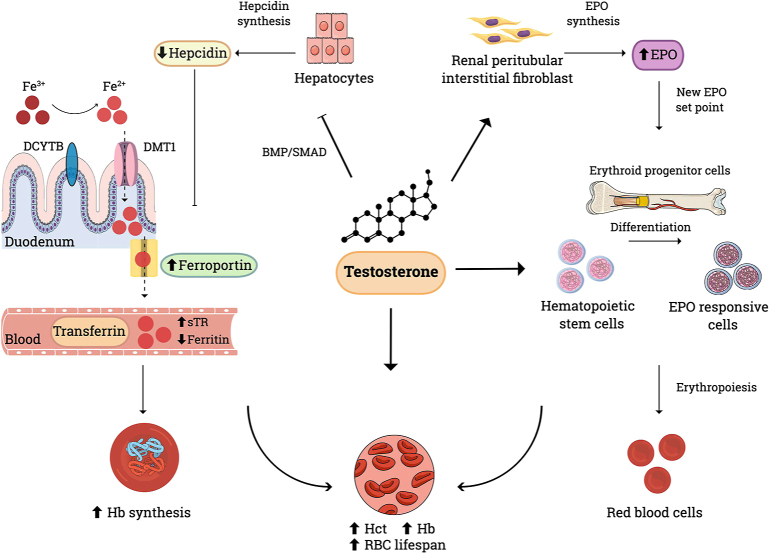
Effects of testosterone on the haematopoietic system. Testosterone promotes RBC synthesis via three principal mechanisms. First, it inhibits hepcidin production by suppressing BMP/SMAD signalling, thereby allowing ferroportin to facilitate intestinal absorption of iron into the bloodstream. This enhances iron availability for Hb synthesis, as indicated by increased levels of sTR and decreased ferritin. Second, it stimulates EPO synthesis in renal interstitial fibroblasts, leading to a transient increase in EPO levels, followed by stabilisation at a new set point concentration relative to Hb levels. Finally, it also enhances the differentiation of haematopoietic stem cells into EPO responsive cells and increases the sensitivity of erythroid progenitor cells to EPO. These actions combined to result in an increased RCM and RBC lifespan. DCYTB: duodenal cytochrome b; DMT1: divalent metal transporter 1; EPO: erythropoietin; Fe^3+^: ferric iron Fe^2+^: ferrous iron; Hb: haemoglobin; Hct: haematocrit; RBC: red blood cells; RCM: red cell mass; sTR: soluble transferrin receptor.

In addition to prolonging RBC lifespan (7), androgens have various other effects on the haematopoietic system, including promoting the differentiation of bone marrow haematopoietic stem cells into EPO-responsive cells (Fig. 2) (27). However, the major androgen-dependent pathway promoting RBC synthesis is enhanced iron utilization. Testosterone increases iron availability by suppressing hepcidin secretion by hepatocytes, which allows ferroportin to facilitate intestinal iron absorption. Increased iron utilization for Hb synthesis is indicated by higher serum levels of soluble transferrin receptor (sTR) and lower levels of ferritin. Testosterone also enhances the sensitivity of erythroid progenitor cells to EPO and stimulates renal EPO secretion, leading to an initial rise in circulating EPO levels. Although EPO concentrations return to baseline within 6 months of starting testosterone treatment, they nevertheless remain comparatively elevated in relation to the new higher Hb and Hct values, suggesting a recalibration of the EPO set point to a higher level in accordance with the new Hb concentration (28, 29).

Conversely, a decline in testosterone levels leads to reduced erythropoietic activity, with both hypogonadism and androgen deprivation associated with lower Hct levels, potentially leading to anaemia, especially in older men. The pattern of Hb decline with androgen-deprivation parallels that of serum testosterone, with a rapid decrease at the initiation of suppression (30, 31). Since oestrogens do not impact erythropoietic parameters, this action of testosterone is necessarily independent of aromatization (30). Supporting the oestrogen-independent action of testosterone on haematopoiesis are the physiological divergence in male and female Hb values from puberty onwards (7); the absence of anaemia in men with rare genetic aromatase or estrogen receptor defects, and the observation that treatment with an aromatas inhibitor, can cause erythrocytosis through increased testosterone levels, as a result of reduced oestradiol-mediated negative feedback on gonadotropin secretion (32).

### SGLT2i and erythropoiesis

**Figure 3:**
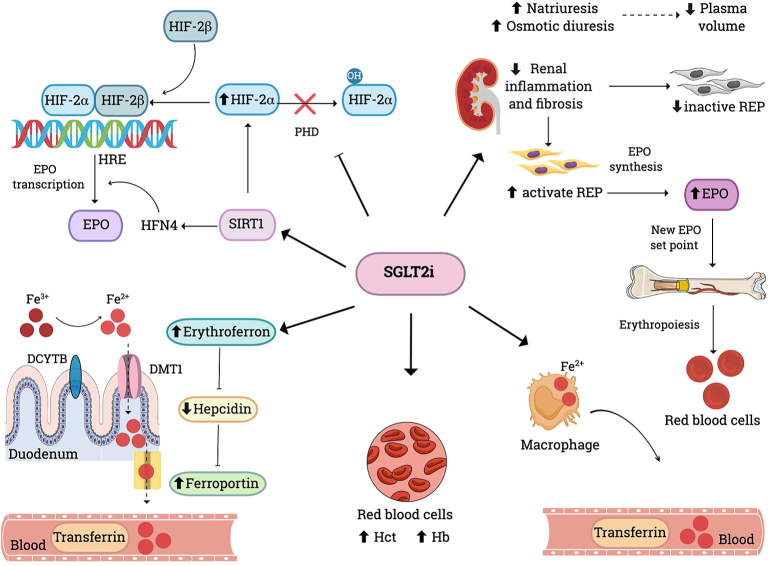
Effects of SGLT2i on erythropoiesis. SGLT2i promote erythropoiesis via multiple mechanisms. First, they increase HIF2α levels by inhibiting PHD enzymes, thereby enhancing EPO transcription. Second, they stimulate SIRT1, which activates HIF2α and HFN4, both of which are involved in EPO synthesis. Third, they suppress hepcidin levels through the induction of erythroferrone, thereby improving intestinal iron absorption and promoting the release of iron from macrophage and cellular storage. Finally, by reducing renal inflammation and fibrosis, they restore REP cells, leading to a transient increase in EPO levels, followed by stabilisation of levels at a new higher set point in relation to RCM. As a result, RBC, Hct and Hb levels all increase. DCYTB: duodenal cytochrome b; DMT1: divalent metal transporter 1; EPO: erythropoietin; Fe^3+^: ferric iron Fe^2+^: ferrous iron; Hb: haemoglobin Hct: haematocrit; HFN4: hepatocyte nuclear factor 4; HIF2α: hypoxia-inducible factor alpha; HIF2β: hypoxia-inducible factor beta; HRE: hypoxia response element; REP: renal erythropoietin-producing cells; RBC: red blood cells; RCM: red cell mass; SIRT1: sirtuin1.

SGLT2i treatment is also associated with a modest but significant increase in Hb levels (6–7 g/L) and Hct (Fig. 3) (16, 33). As with testosterone, the mechanism by which this stimulates erythropoiesis is multifactorial, involving enhanced EPO production and hepcidin suppression, and with only a minor contribution from reduced plasma volume. The reduction in plasma volume (haemoconcentration arising from natriuresis and osmotic diuresis) is, in fact, physiologically transient, and the principal impact of SGLT2i is actually to expand the RCM, evidenced by the onset of reticulocytosis and an early rise in EPO concentrations (19). The rise in EPO levels is likely linked to reduced metabolic stress and is also associated with the increased levels of erythroferrone that suppress hepcidin production in hepatocytes, and thus facilitates iron availability for erythropoiesis (34).

SGLT2i selectively increase levels of HIF2α, which acts as a physiological stimulus for EPO production, while downregulating HIF-1α, which promotes inflammation. This unique action may contribute to their cardio-renal benefits (35). HIF2α connects erythropoietin synthesis to oxygen levels, with its activity regulated by prolyl hydroxylases that facilitate its degradation under sufficient oxygen conditions. The upregulation of HIF2α occurs through the inhibition of these hydroxylases, allowing it to accumulate and boost EPO production. In addition, SGLT2i increase the expression of sirtuin 1 (SIRT1), which may directly or indirectly activate HIF2α and hepatocyte nuclear factor 4 (HFN4), further promoting the transcription of the EPO gene (36). SGLT2i also promote the release of iron from macrophages and intracellular storage sites, and directly enhance intestinal iron absorption (35, 36).

Chronic inflammation and fibrosis in diabetes and CKD may impair EPO production by transforming renal erythropoietin-producing cells (REPs) into non-functional myofibroblasts (34). Patients with T2DM may have lower EPO levels due to metabolic stress in the proximal tubules, which can create a hypoxic environment. These myofibroblasts exhibit decreased sensitivity to hypoxia and produce inflammatory cytokines and fibrotic molecules instead of EPO. SGLT2i mitigate this process by reducing renal inflammation and fibrosis, thus restoring REP functionality and enhancing EPO production (34, 37).

After an initial rise in Hb with SGLT2i treatment, levels may then partially decrease over time, establishing a new set point for EPO that largely avoids the risk of severe erythrocytosis (36). However, in rare instances, SGLT2i may unmask an underlying myeloproliferative neoplasm, which could be seen as a diagnostic benefit (33).

### Erythrocytosis: subtypes, mechanisms and risks

Erythrocytosis is defined as a RCM that is more than 25% above the normal mean value, although it is practically evaluated using Hct. Erythrocytosis can be absolute, due to increased RCM, or relative, due to a reduction in plasma volume, as occurs in states of dehydration or vasoconstriction. However, both absolute and relative erythrocytosis may commonly co-exist due to shared risk factors, such as smoking and hypoxia.

A raised Hct increases blood viscosity which, if combined with stasis and endothelial injury, forms Virchow’s triad, and thus predisposes to hypercoagulability (38, 39). Higher Hct is also linked to reduced venous return and increased platelet adhesiveness. All these mechanisms combine to promote both arterial or venous clot formation, as circulating platelets and coagulation factors locate to the dysfunctional endothelium (39). After adjusting for age, body mass index (BMI) and smoking and excluding those with relevant medical conditions, the Trømso study found a 1.5-fold relative risk of venous thromboembolism (2.4-fold for unprovoked events) in men with Hct in the top quintile (Hct ≥0.46) compared to men in the lowest 40 centiles (39), and the Copenhagen General Population Study found a 1.5-fold relative risk of coronary thrombosis in men in the top 5 centiles (Hct ≥0.48) (38).

Absolute erythrocytosis may be primary or clonal (polycythaemia vera) or secondary. Clonal erythrocytosis is associated with low-normal EPO levels, JAK2 V617F mutation in over 95% of cases, and usually with anomalies of platelets and granulocytes as well as red cells. The risk of thrombosis is particularly high and can be mitigated by antiplatelet drugs and regular venesection (26, 40). In contrast, secondary erythrocytosis usually results from chronic hypoxia or treatment with androgens or SGLT2i, and is characterized by raised EPO levels and negative JAK2 screen. The risk of thrombosis is much lower for the same level of Hct, and accordingly, there is no defined role for venesection.

Relative erythrocytosis, or Gaisbock syndrome, is characterized by elevated Hct with normal RCM, and results from the contraction in plasma volume that is necessarily associated with increased vascular tone. Important associations include hypertension, impaired nocturnal antidiuretic hormone secretion, chronic hypoxaemia, cigarette smoking and alcohol abuse. Regarding hypertension, there is also a positive association between Hb and systolic and diastolic pressure in healthy individuals, with higher Hb being associated with arterial stiffness, nitric oxide (NO) depletion, renin-angiotensin-aldosterone system activation and higher EPO concentrations. However, the exact mechanisms and the direction of causation are uncertain (41). Indeed, there may be bidirectional amplification, or independent causation by unidentified factor(s). Thus, the increased vascular tone associated with chronic hypertension may simply increase Hct through a commensurate contraction in plasma volume (42). Conversely, a higher Hct may raise BP and contribute to the development of hypertension by reducing the availability of NO in vascular smooth muscle, leading to vasoconstriction. Several biological hypotheses have been proposed, including increased blood viscosity and the role of Hb as a scavenger of NO (39).

Although it may be difficult to conclusively differentiate between relative and absolute secondary forms of erythrocytosis, without performing specialized radioisotope-based studies, in practice the two forms frequently coexist due to shared underlying causes, such as cigarette smoking, obesity, sleep apnoea or other cause of chronic hypoxia.

### Is venesection a reasonable treatment for non-clonal erythrocytosis?

Regular venesection is a straightforward technique that quickly reduces Hb and Hct to reference levels and, in JAK2-positive or clonal erythrocytosis, there is good evidence for its safety and efficacy in mitigating the risk of thromboembolic disease. However, in respect of non-clonal erythrocytosis, where the risk of thrombosis is anyway much lower and there is also potential for addressing the predisposing factors, a deeper dive into the evidence becomes necessary.

Repeated venesection can lead to decreased tissue oxygen pressure (pO_2_) and depletion of iron stores, factors which activate biological pathways that may paradoxically promote the risk of thrombosis (43). Indeed, in an observational study of SGLT2i-associated erythrocytosis, thrombosis rates were higher among patients who underwent phlebotomy: 21% (6/29) versus 6% (4/71) (33, 44). A similar pattern was observed in Chuvash erythrocytosis, a congenital disorder where the thrombotic risk is largely independent of Hct and the rate of thrombosis is increased by venesection (45).

Therefore, venesection should not be considered as a primary treatment for non-clonal erythrocytosis. Instead, the management of both absolute and relative forms should primarily focus on lifestyle adjustments: quitting smoking, moderating alcohol and achieving weight loss, treating the associated hypertension relatively aggressively with vasodilator drugs rather than diuretics so as to expand the plasma volume (46), and identifying and treating primary respiratory conditions such as OSA (43). Venesection is thus only considered as a last resort, reserved for only the most severe cases of erythrocytosis and when there are no alternatives, e.g. Hct >0.52 in hereditary or clonal erythrocytosis or >0.56 in COPD (47).

## Testosterone treatment: physiology and clinical implications

### Physiological responses to testosterone treatment

Improvements in sexual interest and energy level and resolution of vasomotor symptoms or mastalgia occur within 3–6 weeks; increased erythropoiesis develops over 2–3 months, and positive changes in lean body mass and bone density over 6–12 months, with the benefits continuing to accumulate for at least another 3 years (1). However, the time needed for testosterone effects to become evident can vary significantly. For instance, in older individuals with erectile or ejaculatory dysfunctions, sexual improvements may take up to 6 months to appear (48).

There is also a dose- and serum level-dependent increase in Hct and Hb with testosterone treatment that is observed in both hypogonadal and transgender males (27, 49, 50). Thus, whilst stimulation of erythropoiesis by testosterone can benefit anaemia, it can also become an adverse effect when there is overshoot resulting in erythrocytosis. Although assessing Hct is arguably the most important aspect of therapeutic monitoring, the risk of thrombosis is more significantly impacted by other vascular risk factors, such as family history, smoking, diabetes mellitus, BMI, hypertension, dyslipidaemia and fibrinogen levels (26, 42). In trans-masculine people, physiological increases in Hb and Hct with testosterone may appear pathological when plotted against female laboratory reference values, and it is suggested that haematological parameters should be evaluated on affirmed gender rather than the sex assigned at birth during hormone treatment (50).

Although older men are more prone to anaemia when hypogonadal, they are at the same time at greater risk of developing androgen-induced erythrocytosis (27, 51). Hence, testosterone therapy is not generally recommended if baseline Hct approaches or exceeds 50%; indeed, such high levels should lead one to seriously question the original diagnosis of hypogonadism (1). Conversely, in older patients with functional CH and anaemia, testosterone treatment corrected baseline anaemia in 40–60% of cases, irrespective of whether another cause of anaemia was identified. These men also experienced improvements in energy and vitality that were not observed in the generality of study patients (49, 52).

In relation to the direct effects of testosterone treatment on BP, oral preparations may cause a very small increase (53), with other formulations not showing any increase at physiological doses.

### Testosterone formulations and their relationship to the haematocrit

Long-acting testosterone undecanoate IM injections maintain more stable serum testosterone levels compared to shorter-acting one, avoiding peaks. In contrast, short-acting testosterone IM injections cause greater fluctuations in testosterone levels, with rapid supraphysiological peaks shortly after injection, followed by a drop to low levels just before the next injection. These fluctuations appear to be associated with an increased risk of erythrocytosis. Transdermal formulations achieve more stable levels, and potentially, a lower overall area-under-curve serum testosterone concentration (1, 54). In cases of clinically significant erythrocytosis with no other risk factors to address (smoking, obesity, hypertension, sleep apnoea, etc.), it is recommended to reduce the testosterone dose and/or switch to transdermal formulations. However, one study found that transgender men on testosterone undecanoate depot injections had lower erythrocytosis rates than those on gels or intermediate-acting IM testosterone esters (9.2% Hct >50 vs 12.8% and 15.9%, respectively), emphasising the relatively greater importance of the overall area-under-curve dose over the actual mode of delivery (54).

### Cardiovascular safety of testosterone

The potential consequences of erythrocytosis due to testosterone supplementation include adverse CV effects, although adaptive mechanisms might reduce these risks, as evidenced by the reported low rates of cerebrovascular events (28). Physiologically, testosterone induces acute coronary vasodilatation, as per *in vitro* and animal models, and also increases coronary blood flow in humans (55). This effect is independent of the vessel endothelium, but it is associated with the activity of ion (particularly potassium and calcium) channels (55, 56). In addition, testosterone improves vascular reactivity, increases muscle mass, reduces overall and visceral fat mass and shortens QTc interval. However, it can also reduce high-density lipoprotein cholesterol, promote platelet aggregation by thromboxane A2 stimulation and cause sodium and water retention (10). Historical retrospective cohort studies of men receiving testosterone treatment found conflicting results regarding CV risk, with some indicating increased risk, others a reduced risk and some showing no effect (11).

Nevertheless, recent prospective studies have found no evidence that testosterone treatment modifies CV risk in the short-to-medium term in older men with functional CH, although the longer-term safety/benefit profile is less certain (10, 11). In men with hypogonadism and established CVD or multiple risk factors for cardiac events, testosterone treatment was found to be non-inferior to placebo in relation to the occurrence of major adverse cardiovascular events (MACEs) during a mean 22-month follow-up (11). Moreover, an individual patient data meta-analysis of 3,600 trial patients did not identify any patient characteristics that significantly modified the risk of incident CVD during treatment (10). Although the TRAVERSE study reported a low incidence of adverse effects, some events occurred with a higher incidence (e.g. pulmonary embolism, non-fatal arrhythmia, atrial fibrillation, acute renal failure and non-fragility fractures), highlighting the need for prescribing caution in men with functional CH (11, 57).

Men with Klinefelter syndrome have an increased risk of metabolic syndrome and CVD, and up to six-fold increased risk of venous thromboembolism, likely arising from the over-expression of clotting factors VIII and IX due to the additional X-chromosome. However, population registries have not observed any modification of these risks according to whether or not they were receiving testosterone treatment (58). Most data on the cardiovascular safety of testosterone therapy derive from studies considering functional CH, making it difficult to draw conclusions for pathological hypogonadism (59). However, recent Danish registry data found a lower all-cause mortality in Klinefelter men receiving testosterone treatment compared with those untreated (60).

## Conclusions

As the clinical use of SGLT2i continues to expand and the recognition and treatment of male hypogonadism increases, clinicians will increasingly find themselves managing patients receiving both SGLT2i and testosterone treatments. Co-prescribing is likely to occur in i) older men with functional CH and T2DM related to obesity, ii) men with advanced CKD and pathological hypogonadism and iii) transgender males of all ages with T2DM, CKD or CVD. A recent large cohort study found that the combined use of SGLT2i significantly increased the relative risk of developing new onset erythrocytosis compared to either treatment prescribed alone. However, the absolute risk of developing erythrocytosis was low even with combination therapy. Nevertheless, careful monitoring of Hct in these men is advised, especially during the first year of therapy, alongside carefully addressing other risk factors (16).

A significant proportion of men with CKD (up to 70%) experience symptoms or signs of hypogonadism, with stages 4–5 CKD characterised by decreased bone density, muscle wasting, low libido and anaemia. Testosterone therapy can alleviate these symptoms and provides additional benefits compared to ESAs, and at a much lower cost. For a man with pathological hypogonadism, stage 4–5 CKD and osteoporosis, the safest treatment for bone protection is arguably transdermal testosterone (67). Such a treatment strategy is also likely to greatly reduce the need for more expensive ESAs, and especially if combined with SGLT2i. However, in older men with mild functional CH due to obesity, testosterone treatment is associated with an excess of non-fragility fractures (57) with a putative behavioural basis, despite otherwise improving all measurable aspects of bone structure and health (61, 62).

Guidelines from the Society for Endocrinology emphasise the need to optimise therapy while avoiding both under- and over-treatment with testosterone. It is important to highlight to patients the benefits of testosterone treatment on bone, muscle and Hct, in addition to the more familiar benefits to sexual function. These effects, along with improvements in psychological well-being, contribute to a better quality of life for men with pathological hypogonadism undergoing lifelong testosterone treatment. A symptom diary can help identify the side effects and facilitate treatment adjustments based on biochemical data, but maintaining Hct in the safe range should be the overriding aim (1). The rationale for testosterone treatment of men with functional CH due to obesity is less certain, especially in relation to fracture prevention (57), but men with baseline anaemia do seem to derive the greatest overall symptomatic benefit (49, 52).

Clinicians should also be aware of sex differences that influence hypertension, obesity, and diabetes prevalence and severity. Although masculinising hormone treatment of transgender individuals notably impacts surrogate markers of CV risk, these changes do not seem to directly translate into a measurable increase in CV events (63, 64). Effective monitoring and management of risk factors such as alcohol consumption, smoking, high BP and elevated BMI can help mitigate potential CV risks associated with testosterone therapy in both hypogonadal and trans-masculine individuals.

Both androgens and SGLT2 inhibitors can induce erythrocytosis via similar mechanisms. A cohort study of SGLT2i-induced erythrocytosis found a 10% incidence of arterial and venous thrombotic events among patients treated over a median of 24 months, compared with the expected 0.2–0.4% incidence of venous thrombosis over 2 years in European and general population, albeit this varies according to age, sex, lifestyle and comorbidities; diabetes increase the risk by around 50% (65). However, no association of thrombosis risk was found with basal or peak Hct (44, 66).

In shared decision-making, a one-off therapeutic venesection should be considered only as a very last resort treatment for non-clonal erythrocytosis when other options are not feasible, due to the uncertainties surrounding its efficacy and safety and, indeed, the potential for promoting thrombosis. Lowering the testosterone dose (with a switch to transdermal) and targeting modifiable risk factors (smoking-cessation, weight loss, BP lowering with ACE-inhibitor or ARB drugs, identification and treatment of OSA) are the mainstays of management. However, persisting erythrocytosis due to SGLT2i (even after addressing all other modifiable risk factors) should probably be tolerated due to the other major cardiometabolic benefits. Regular prophylactic venepuncture (or covert blood donation) is explicitly discouraged unless clonal erythrocytosis arising from JAK2-mutation was unexpectedly identified (43, 44).

In patients experiencing secondary erythrocytosis while receiving both SGLT2i and testosterone, clinicians should i) determine just how sound was the original indication for testosterone therapy (e.g. survivor of childhood cancer versus obese man with mild sexual symptoms and slightly low T level without anaemia or raised LH), ii) evaluate and manage all modifiable risk factors, which should include the aggressive treatment of incident hypertension with vasodilator drugs, and iii) adjust the dose and delivery route, before considering complete discontinuation of treatment. Adjustments in dosage or formulation may help reduce thrombotic risk while maintaining the therapeutic benefits of these treatments.

When testosterone is started for a verified diagnosis of pathological hypogonadism, it is anticipated to be continued lifelong under most circumstances. By properly monitoring treatment, adjusting the dose or dose-interval accordingly, collaboratively addressing other risk factors, it should usually be possible to avoid inducing erythrocytosis in the first place. When erythrocytosis does occur, then this can usually be addressed by following the same paradigm, rather than complete interruption of treatment. On the positive side, testosterone therapy in combination with SGLT2i potentially represents a cheaper and safer alternative to ESAs in men with advanced CKD and PH (which is likely underdiagnosed). Given the significant risks and uncertain benefits of conventional bone-specific drugs in men with advanced CKD, this may be especially important for those men with associated osteoporosis (67).

## Declaration of interest

RQ received Advisory Board feed from Roche Diagnostics (2023) and Besins (2024), and a speaker’s honorarium from Androlabs (2025).

## Funding

This work did not receive any specific grant from any funding agency in the public, commercial or not-for-profit sector.

## Ethics

No ethics or IRB approval was required or sought, as this review involved no original or unpublished research.
